# Magnetic Resonance Imaging Findings of an Intrahepatic Bile Duct Adenoma: A Case Report

**DOI:** 10.7759/cureus.27082

**Published:** 2022-07-20

**Authors:** Jie Yuan, Kun Liu, Mengxiao Liu, Songhua Zhan

**Affiliations:** 1 Department of Radiology, Shuguang Hospital Affiliated to Shanghai University of Traditional Chinese Medicine, Shanghai, CHN; 2 Department of Pathology, Shuguang Hospital Affiliated to Shanghai University of Traditional Chinese Medicine, Shanghai, CHN; 3 Magnetic Resonance Scientific Marketing and Diagnostic Imaging, Siemens Healthineers Ltd., Shanghai, CHN

**Keywords:** diffusion-weighted imaging, gd-eob-dtpa, gadolinium ethoxybenzyl diethylenetriaminepentaacetic, magnetic resonance imaging, bile duct adenoma

## Abstract

Bile duct adenoma (BDA) is a benign tumor that arises from the epithelium of the intrahepatic bile ducts. Herein, we present a case and discuss the characteristic magnetic resonance imaging (MRI) features of intrahepatic BDA by radiologic-pathologic correlation. A 41-year-old male visited our hospital. He was incidentally shown to have a liver-occupying lesion during a routine medical examination. MRI revealed a 16 mm × 17 mm × 18 mm circular hepatic mass occupying segment 2 of the liver. It showed low signal intensity on T1-weighted images (T1WI) and high signal intensity on T2-weighted images (T2WI). Diffusion-weighted imaging (DWI) MRI showed a ring of high intensity. Gadolinium ethoxybenzyl diethylenetriaminepentaacetic (Gd-DTPA) dynamic enhanced scanning showed a prolonged “ring enhancement” pattern. It showed a ring of high intensity in the hepatobiliary specific period and low signal peripheral and central of the tumor. The pathology result of the surgical resection showed a diagnosis of intrahepatic BDA. Postoperatively, the patient is currently under outpatient observation for seven months with no apparent recurrence. Intrahepatic BDA can be characterized as a small circular lesion located in the liver. MRI and pathologic features are well characterized in this tumor. MRI enhancement plays an important role in the diagnosis and evaluation of BDA.

## Introduction

Intrahepatic bile duct adenoma (BDA) is a rare benign epithelial hepatic tumor arising from bile duct cells [1]. It represents about 1.3% of primary hepatic tumors; intrahepatic BDA is often an incidental finding in diagnostic imaging or identified in the evaluation of nonspecific symptoms [2]. A definitive diagnosis is based on pathologic findings. It is sometimes difficult to make an accurate diagnosis before surgery. In this study, we describe the imaging findings of intrahepatic BDA using magnetic resonance imaging (MRI) and compare the imaging and pathologic findings of BDA.

## Case presentation

A 41-year-old male visited our hospital and was incidentally found to have a space-occupying lesion in the liver during a routine medical examination. No additional disease was found in the patient’s medical history. His serum alpha-fetoprotein (AFP) was 1.95 ng/mL (normal range: 0-8.78 ng/mL), and his carbohydrate antigen 199 was 7.58 U/mL (normal range: 0-39 U/mL).

MRI of the upper abdomen was performed using a 3.0-Tesla MRI scanner (Siemens, Erlangen, Germany). Axial T1-weighted imaging (T1WI), T2-weighted imaging (T2WI) with fat saturation, in-phase and out-phase images, diffusion-weighted imaging (DWI) (b-value: 0; 800 seconds/mm^2^), and dynamic contrast enhancement were used after gadolinium ethoxybenzyl diethylenetriaminepentaacetic (Gd-DTPA) acid was injected manually at 0.2 mL/kg body weight, and gadolinium ethoxybenzyl diethylenetriaminepentaacetic (Gd-EOB-DTPA) acid-enhanced MRI was also performed. The hepatobiliary phase was obtained 25 minutes after the injection of Gd-EOB-DTPA.

**Figure 1:**
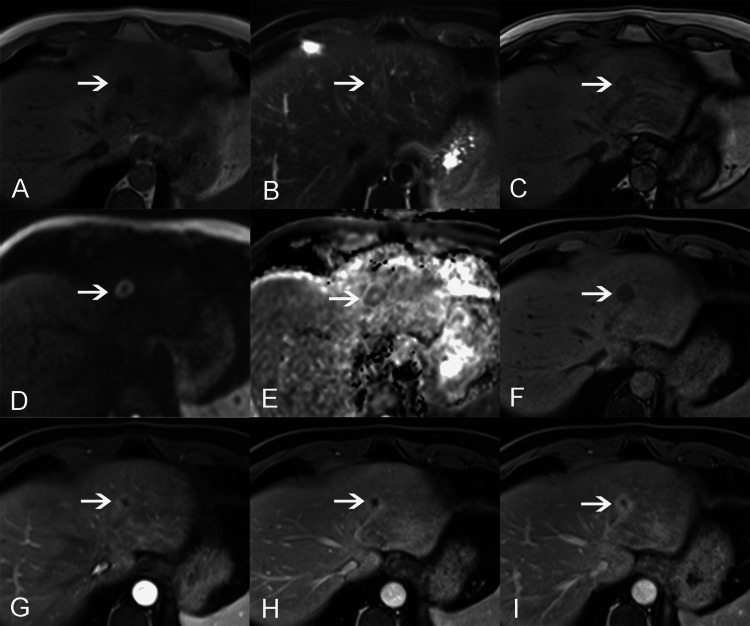
MRI findings. MRI revealed a round mass (arrows) with a clear edge in segment 2 of the liver. It showed low signal intensity on T1WI and high signal intensity on T2WI (A,F,B). The fat component was not demonstrated (C). DWI MRI showed a ring of high intensity (D). The rim of the lesion appeared slightly hypointense to the surrounding parenchyma on ADC maps (E). Gd-DTPA dynamic enhanced scanning showed a prolonged “ring enhancement” pattern (G-I).

MRI revealed a 16 mm × 17 mm × 18 mm mass with a clear edge in segment 2 of the liver. It showed low signal intensity on T1WI and high signal intensity on T2WI (Figure 1A, 1B, 1F). The fat component was not demonstrated with chemical shift imaging, and the lesion remained hypointense (Figure 1C). DWI MRI showed restricted diffusion along the rim (Figure 1D). On the apparent diffusion coefficient (ADC) maps, the lesion appeared slightly hypointense to the surrounding parenchyma with an ADC value calculated as 591/mm^2^ (Figure 1E). Gd-DTPA dynamic enhanced scanning showed a prolonged “ring enhancement” pattern (Figure 1G-1I). MRI also showed a blood vessel that extended to the ventral side of the mass (Figure 1I).

**Figure 2:**
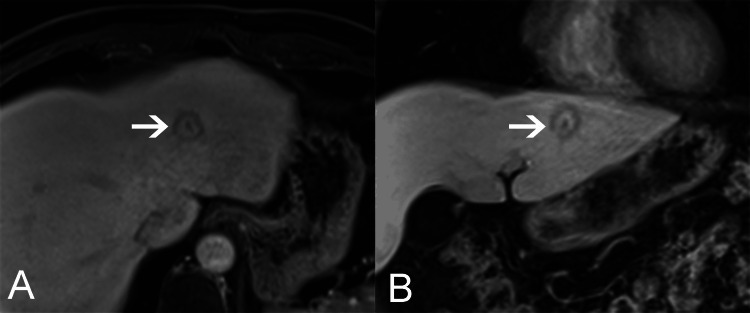
Gd-EOB-DTPA-enhanced MRI findings of BDA in axial (A) and coronal (B) views. The tumor showed a ring of high intensity (arrows) in the hepatobiliary specific period and low signal peripheral and central of the tumor.

There was no pseudo-capsule. It showed a ring of high intensity in the hepatobiliary specific period and low signal peripheral and central of the tumor (Figure 2A, 2B).

**Figure 3:**
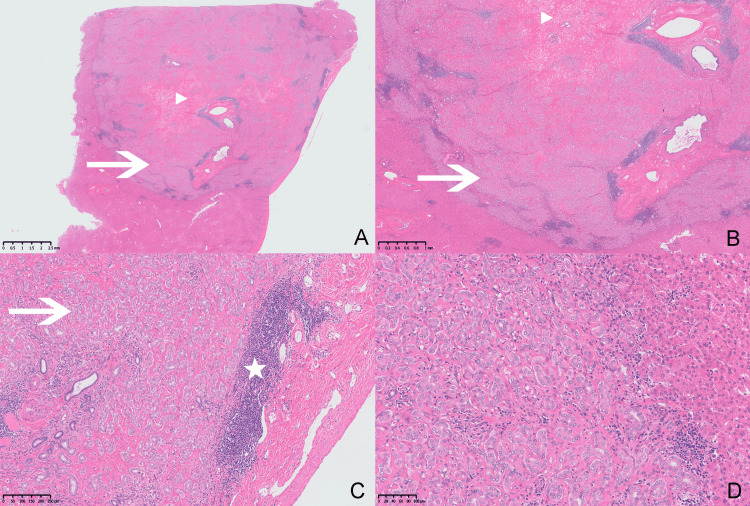
Histopathologic findings. (A) A relatively circumscribed nodular proliferation of well-formed ducts by cytologically bland cuboidal epithelial cells (arrow). (B-D) Glandular epithelial cells that were cuboid, and nuclei are round and oval. There are many fibrous hyperplasias and hyaline degeneration in the center of the mass (arrowheads). The lesion comprised inflammatory cell infiltration (pentagram).

Partial resection of the left lateral lobe of the liver was performed. The gross of the resected tumor appeared as a clear border, moderate hardness, and ashen round tubercle. Microscopy revealed a relatively circumscribed nodular proliferation of well-formed ducts by cytologically bland cuboidal epithelial cells (Figure 3A). Glandular epithelial cells are cuboid, and nuclei are round and oval (Figure 3B-3D). The morphology of the nucleus is consistent, and the cytoplasm is weakly stained. Mitotic activity was inconspicuous. A prominent lymphoid infiltrate forming reactive follicles with germinal centers was observed in the glandular ducts. There is no saccular dilation of the glandular ducts. There are many fibrous hyperplasias and hyaline degeneration in the center of the mass (Figure 3A, 3B). The boundary between the tumor and the surrounding normal liver was not clear, and no compression was observed in the normal liver.

After seven months of follow-up, no evidence of recurrence was found.

## Discussion

Intrahepatic DBA is a rare form of benign tissue neoplasm that arises from the epithelial cells of the liver. DBA is found in approximately 1.3% of primary hepatic tumors [2]. They were usually subcapsular, were single, ranged in size from 1 to 20 mm (mean: 5.8 mm), and were well circumscribed but nonencapsulated. They had limitations on growth potential. Clinically, most patients with DBA are asymptomatic, and DBA is incidentally discovered. Our patient was a middle-aged male, who was also found to have a hepatic tumor incidentally during a routine medical examination.

Histologically, BDA is composed of small-sized tubules with a definite lumen of cuboidal cells that have uniform round nuclei. It has varying degrees of fibrosis and inflammation and has no cellular atypia or mitotic activity [3]. Immunohistochemical staining for CK7, CK19, CK10, and CD56 are positive, whereas for p53 and AFP are negative in BDA.

In MRI, intrahepatic BDAs appear as low or slightly low signal on T1WI and high or slightly high signal on T2WI, and some showed a partial ring of high signal [4]. In DWI, they show high or slightly high, and some showed a ring of high signal. BDA demonstrated two main ways on dynamic enhanced MRI [5]: moderate or obvious ring enhancement was seen in the arterial phase, and continuous enhancement was seen in the portal phase and delayed phase. In the delayed phase, the tumor showed low signal intensity in the center and high signal intensity in the periphery. The continuous enhancement was related to the presence of more fibrous tissue in the tumor. The hepatobiliary specific period of Gd-EOB-DTPA-enhanced examination showed that the lesions displayed a low signal in previous cases [5]. However, in our case, the central area of the BDAs was hypointense compared with the surrounding hyperintense in the hepatobiliary phase. The peripheral area of the lesion, which consisted of densely packed simple tubular bile ducts, was hyperintense. That was because Gd-EOB-DTPA filled the saccular dilated bile duct. A hypointense band at the center areas can be observed, which consisted of many fibrous hyperplasias and hyaline degeneration.

Intrahepatic BDAs need to be differentiated from focal nodular hyperplasia (FNH), hepatocellular adenoma (HCA), hepatic neuroendocrine tumor (PHNET), hepatocellular carcinoma (HCC), metastatic disease, and cholangiocarcinoma. The signal intensity of FNH on MRI is close to that of the normal liver parenchyma on T1WI and T2WI, which usually has starlike scars in the middle of the lesion. It has a delayed enhanced signal intensity and takes up Gd-EOB-DTPA [6]. The lack of a typical MRI finding makes it difficult to differentiate HCA from FNH. It is usually composed of various hemorrhages, fats, and necrosis. It appears hypointense in the hepatobiliary phase [7]. PHNET shows marked enhancement in the arterial phase, but it shows a washout in the portal venous phase and higher enhancements in the delayed phase compared to the surrounding hepatic parenchyma [8]. HCC is usually associated with clinical backgrounds including hepatitis and cirrhosis with an elevated serum AFP. The dynamic MRI scan shows arterial hyperenhancement with “washout” in the portal venous or delayed phase. [9]. Metastatic tumors have a history of malignancy in other parts of the body. They often appear as multiple, varying in size and annular enhancement [10]. Intrahepatic cholangiocarcinoma generally shows uneven signal and dilatation of the intrahepatic bile duct with an elevated serum carbohydrate antigen 19-9. It takes up contrast agents progressively during arterial and venous phases [11].

Intrahepatic BDA is considered benign, but a previous study found that BDA may be precursor lesions of small duct intrahepatic cholangiocarcinoma [12]. Thus, regular follow-up is still recommended.

## Conclusions

In conclusion, the present report describes the MRI features of an intrahepatic BDA, and MRI and pathologic features are well characterized in this tumor. Although the final diagnosis still depends on pathology due to its rarity, MRI enhancement still plays an important role in the diagnosis and evaluation of BDA. Considering that some BDAs may be precancerous of cholangiocarcinoma, follow-up is still needed.
